# Supplementary figures and images for: Ventricular divergence correlates with epicardial wavebreaks and predicts ventricular arrhythmia in isolated rabbit hearts during therapeutic hypothermia
Source: PLoS One. 2020 Feb 21;15(2):e0228818. doi: 10.1371/journal.pone.0228818 (PMC7034916; doi:10.1371/journal.pone.0228818)

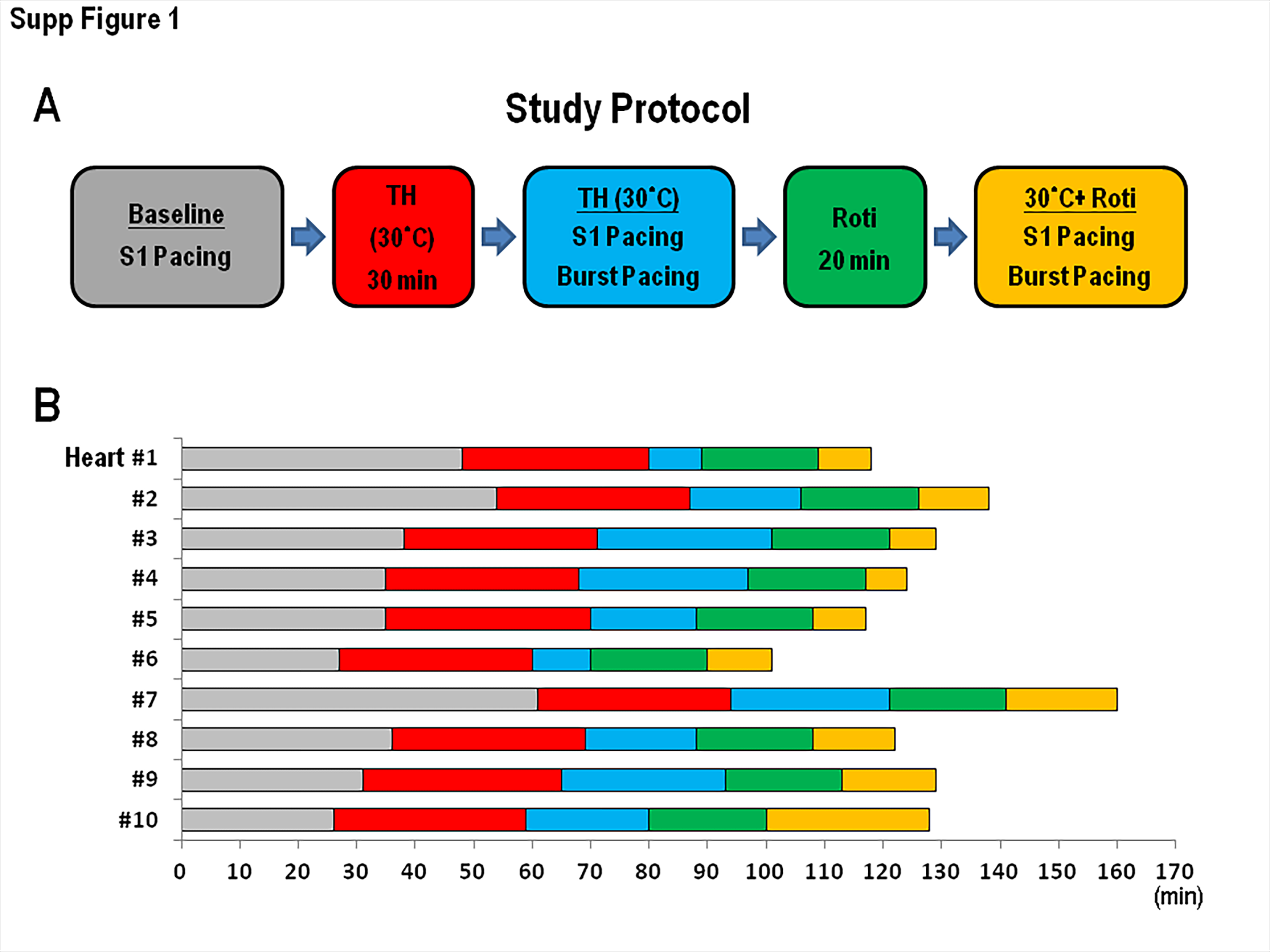

Supplement: S1 Fig — (TIF) [file pone.0228818.s001.tif]
