# Supplementary material for: Ventricular divergence correlates with epicardial wavebreaks and predicts ventricular arrhythmia in isolated rabbit hearts during therapeutic hypothermia
Source: PLoS One. 2020 Feb 21;15(2):e0228818. doi: 10.1371/journal.pone.0228818 (PMC7034916; doi:10.1371/journal.pone.0228818)
Supplement: S1 Table — (DOCX) [file pone.0228818.s002.docx]

S1 Table. Pacing cycle lengths and results of each attempt before and after rotigaptide treatment during hypothermia (30°C)

|  |  |  | Hypothermia (30°C) | | | | |  |  |  |  |  | Hypothermia (30°C) + Rotigaptide | | | | |  |  |  |
| --- | --- | --- | --- | --- | --- | --- | --- | --- | --- | --- | --- | --- | --- | --- | --- | --- | --- | --- | --- | --- |
| Pacing attempts | #1 | #2 | #3 | #4 | #5 | #6 | #7 | #8 | #9 | PIVF  % | #1 | #2 | #3 | #4 | #5 | #6 | #7 | #8 | #9 | PIVF  % |
| Heart #1 | 240  NVF | 230  NVF | 230  NVF | 140  NVF | 150  NVF |  |  |  |  | 0% | 200  NVF | 180  NVF | 140  NVF | 120  NVF |  |  |  |  |  | 0% |
| Heart #2 | 180  VF | 200  NVF | 180  VF | 160  VF | 130  VF | 260  VF | 260  VF | 180  VF | 280  VF | 89% | 200  NVF | 190  NVF | 190  NVF |  |  |  |  |  |  | 0% |
| Heart #3 | 230  NVF | 230  NVF | 230  NVF | 260  VF | 260  VF |  |  |  |  | 40% | 220  NVF | 230  NVF | 310  NVF |  |  |  |  |  |  | 0% |
| Heart #4 | 190  VF | 190  VF | 190  NVF | 230  NVF | 200  NVF |  |  |  |  | 40% | 180  NVF | 190  VF | 190  VF | 180  NVF | 180  NVF |  |  |  |  | 40% |
| Heart #5 | 180  VF | 180  NVF | 180  VF | 190  VF | 190  VF | 190  VF | 190  NVF | 180  VF | 180  NVF | 67% | 190  NVF | 190  NVF | 190  NVF | 180  NVF |  |  |  |  |  | 0% |
| Heart #6 | 260  NVF | 270  NVF | 240  NVF | 240  NVF | 240  NVF |  |  |  |  | 0% | 300  NVF | 300  NVF | 300  NVF | 300  NVF |  |  |  |  |  | 0% |
| Heart #7 | 180  VF | 200  VF | 200  VF | 200  NVF | 200  VF | 200  VF | 180  VF | 180  VF | 180  VF | 89% | 240  VF | 200  NVF | 190  VF | 200  VF | 200  VF | 180  VF | 200  NVF | 200  VF | 200  VF | 78% |
| Heart #8 | 140  VF | 140  NVF | 130  VF | 140  NVF | 140  VF |  |  |  |  | 60% | 180  VF | 150  NVF | 140  NVF | 130  NVF | 130  VF | 150  VF | 150  VF | 180  VF | 180  VF | 67% |
| Heart #9 | 230  NVF | 230  NVF | 230  VF | 230  VF | 240  VF |  |  |  |  | 60% | 230  NVF | 230  NVF | 240  VF | 230  NVF | 240  VF |  |  |  |  | 40% |
| Heart #10 | 260  VF | 240  VF | 240  NVF | 280  NVF | 240  NVF |  |  |  |  | 40% | 240  NVF | 240  NVF | 240  NVF | 240  NVF |  |  |  |  |  | 0% |

VF, ventricular fibrillation; NVF, no VF; PIVF, pacing-induced VF.
