# Supplementary material for: Ventricular divergence correlates with epicardial wavebreaks and predicts ventricular arrhythmia in isolated rabbit hearts during therapeutic hypothermia
Source: PLoS One. 2020 Feb 21;15(2):e0228818. doi: 10.1371/journal.pone.0228818 (PMC7034916; doi:10.1371/journal.pone.0228818)
Supplement: Attachment — Submitted filename: Response to Reviewers 0121.docx [file pone.0228818.s006.docx]

MS Number: PONE-D-19-26993R2

Responses to Editor

Comments to the Author

The authors, overall, have addressed most of the concerns raised by the reviewers. The reach of the conclusions based on the experimental model used, has been toned down. Furthermore, new experimental series (II, III and IV), additional results and modifications of the already presented, were added, which have contributed to improve the paper from its previous version, thus addressing methodological questions that could limit the validity of the results. The number of experiments in protocol III could have been higher, since n=2 hearts seems a bit low in order to perform statistical analyses (even with non-parametric tests, and even using several recordings per heart). On the other hand, those aspects that could not been addressed with experimental data, were included as limitations.

**Response:**

We thank the editor for these insightful comments. In protocol III, we have performed an experiment to increase the heart number (n=3) and improve statistical analyses. (p 7, para 3)

The addition of this new data did not change the main findings in protocol III as below:

“**Different Pacing Sites on the Divergence and VF Inducibility**

The divergence of the PIVF episodes was not different between RV (0.15±0.04) and LV (0.15±0.07) pacing (p=0.97). Also, the divergence of the non-PIVF episodes was not different between RV (0.12±0.07) and LV (0.11±0.05) pacing (p=0.60). The inducibility of VF was also similar before (p=0.20) and after (p=0.23) rotigaptide infusion between RV and LV pacing. These findings indicated that the divergences and VF inducibility in this model were not affected by changing the pacing sites.” (p 14, para 1)

I have, however, some minor concerns:

1. I would suggest removing the sentence in Methods (page 10, paragraph 2 "To achieve a medium effect size of 0.15 and a power of 0.8 in linear regression, the predictive sample size is 55".), since it is already clear that the model does not provide high predictive power with the determination coefficient (R2), as it is also stated in the text.

**Response:**

We have removed this sentence in the method section. (p 10, para 2)

2. The authors should review the text and correct text editing (i.e. page 7 last line, should read “H₂O” with 2 in subscript instead of “H2O”; p13, par2 should read “...and fibrillatory conduction (movie S1).” instead of “…and fibrillatory conduction. (Supp movie 1)”; p14 para1 “R²” with 2 in superscript instead of “R2”, among many others).

**Response:**

We have corrected the text editing problems, including those mentioned by the editor, throughout the manuscript. (highlighted parts)

3. Referencing should be revised, the number in brackets should be written before the period “[1].” And not after “.[1]”

**Response:**

We have moved the “number in brackets” to be before the period throughout the manuscript.

4. Modify the sentence in p17 par1 to “These findings suggest that ventricular divergence on ECG could be a predictor of epicardial wavebreaks during PVS”. P17 par2 revise the meaning of “In multiple wavelet hypotheses, wavebreaks resulting from collisions of propagation wavelets and generations of new wavelets are the basis for VF maintenance.[27] Using optical mapping techniques, PS found on phase maps is the area of ambiguous activation state which underlies the formation of rotor and wavebreaks which serve as a source of VF.[10]”.

**Response:**

We have modified this sentence as the editor’s suggestion as “These findings suggest that ventricular divergence on ECG could be a predictor of epicardial wavebreaks during PVS.” (p 17, para 1)

The meaning of “multiple wavelet hypothesis” has also been revised as “In multiple wavelet hypothesis, wavebreaks resulting from collisions of propagation mother wavelets might generate daughter wavelets in a self-sustaining turbulent process to maintain VF [27]. Using optical mapping techniques, PS found on phase maps is the area of ambiguous activation state which underlies the formation of rotor and wavebreaks, and serves as a source of VF [10].” (p 17, para 2)

5. Soften the conclusion regarding the predictive value of ventricular divergence, i.e. “Ventricular beat-to-beat morphological variation evaluated as divergence was significantly correlated with, and could predict epicardial wavebreak during ventricular pacing at TH” in the abstract and conclusion sections.

**Response:**

We have revised the conclusions in the abstract as “Ventricular divergence correlated with, and might be predictive of epicardial wavebreaks during PVS at TH.” (p 2, abstract)

Also, we softened the main conclusion in the manuscript as “Ventricular beat-to-beat morphological variation evaluated as divergence was correlated with, and might be predictive of, epicardial wavebreak during ventricular pacing at TH.” (p 20, para 2)

6. Remove “*Fisher’s exact test” from Fig 2 and include the information in Statistical Analysis.

**Response:**

We have removed the “*Fisher’s exact test” from Fig 2, and included the information in Statistical Analysis as “Fisher’s exact test was used to compare the categorical data between groups.” (p 10, para 2)
